# Job Insecurity in the COVID-19 Pandemic on Counterproductive Work Behavior of Millennials: A Time-Lagged Mediated and Moderated Model

**DOI:** 10.3390/ijerph18168354

**Published:** 2021-08-06

**Authors:** Fei Yiwen, Juhee Hahn

**Affiliations:** 1 The Graduate School, Chung-Ang University, Seoul 06974, Korea; dengdao2569@naver.com; 2 Department of Business Management, Chung-Ang University, Seoul 06974, Korea

**Keywords:** job insecurity, negative emotions, moral disengagement, counterproductive work behavior, psychological capital, millennials

## Abstract

During the COVID-19 pandemic, the market environment for the information technology (IT) industry changed dramatically, presenting companies with numerous obstacles in day-to-day management activities and changing business needs. Previous studies found that job insecurity due to COVID-19 significantly impacted millennials. Our research explored the effect of job insecurity on counterproductive work behavior among millennial employees during the COVID-19 period, using moral disengagement as a mediating variable, and psychological capital and negative emotions as moderating variables. In this study, 298 employees working in Chinese IT companies completed the questionnaire survey. We collected data from employees over three different time intervals (baseline, three weeks later, and six weeks later) to mitigate the issues of common method bias and single-source data. We analyzed the collected data using SPSS25.0 and Amos24.0 for structural modeling. Our research results indicate that job insecurity is positively associated with counterproductive work behavior, and moral disengagement plays a mediating role. In addition, psychological capital moderates the relationship between job insecurity, moral disengagement, and counterproductive work behavior. Negative emotions also moderate the mediating effect of moral disengagement between job insecurity and CWB.

## 1. Introduction

The outbreak of coronavirus disease (COVID-19) placed a significant restraint on the information technology market, as trade restrictions disrupted global supply chains and slowed production. The resulting business reduction forced some companies to the verge of bankruptcy, necessitating staff layoffs for business survival. For some Internet companies, layoffs were the most direct and necessary step for closing the expense–income gap. As a result, it appears improbable that employees would not care about their employment conditions due to COVID-19 [1]. Jung et al. [1] note that job insecurity due to COVID-19 has a more substantial impact on millennials. According to China′s National Bureau of Statistics, in 2020, Chinese millennials were employed in 53% of companies and comprised more than half of the total number of employees in companies. Yunita and Saputra [2] found that Chinese millennials were born during a period of rapid economic development and received a better education than previous generations. The authors noted that millennials can learn and think uniquely about the workplace, but are vulnerable to hardship and stress. The study also stated that if millennials encounter difficulties at work and cannot obtain help, they are more likely to experience negative emotions and lose enthusiasm for their work.

Counterproductive work behavior (CWB) has increasingly attracted a significant amount of attention from researchers as an invisible but persistent organizational problem. Cases of serious business consequences resulting from CWB are common. However, recent research has focused on traditional employees in traditional industrial settings, ignoring the most recent characteristics among knowledge workers in the new economy [3]. Moreover, employees in the information technology industry, who often have access to tools that are critical for business growth or innovation, may be more at risk for CWB. Huang et al. [4] found that job insecurity influences counterproductive work behaviors by increasing moral disengagement; that is, job insecurity may cause employees to create rationalizations that mitigate the cognitive dissonance produced by their transgressions. Their research focused on the consequences of moral disengagement but not the deterring factors. Therefore, our research introduced psychological capital as a positive resource, and explored whether it can mitigate the adverse effects of job insecurity and moral disengagement, among others, and improve employee performance.

Our study′s purpose was to explore the effect of job insecurity on counterproductive work behavior among millennial employees during the COVID-19 period, using moral disengagement as a mediating variable, and psychological capital and negative emotions as moderating variables.

## 2. Hypotheses Development

### 2.1. Job Insecurity and CWB

As a result of COVID-19, the labor market has changed dramatically, and the issue of job instability has recently received a significant amount of attention. Greenhalgh and Rosenblatt [5] conceived of the term “job insecurity.” Job insecurity refers to a person′s doubt about their job′s long-term viability. It encompasses not only the loss of a job, but also other key job features [6]. Many academics have endorsed and supported this theory. Greenhalgh and Rosenblatt [5] investigated four critical elements of job insecurity after conducting additional studies on the subject: (1) the expectation of consistency when exposing employees to numerous insecure conditions; (2) the threat faced by employees who perceive a violation of their hopes of maintaining stability; (3) job characteristics at risk, in which employees feel threatened by specific job characteristics, such as changing managers, going to a location they do not like, and performing a job they do not like; and (4) powerlessness, i.e., employees who do not feel secure often feel weak, and job insecurity will not arise if employees can resist this emotion when threatened.

Any purposeful activity by a member of an organization, which the organization sees as adverse to its legitimate interests and harmful to its members, is counterproductive to work behavior [7]. According to Hollinger and Clark [8], there are two types of counterproductive work behaviors. The first is “property deviance”, which involves misusing employer assets, theft, property destruction, and the abuse of discount advantages. The second type is “production deviance”, which consists of breaking work process rules. This rule-breaking includes absenteeism, tardiness, long pauses from work, and habits that detract from productivity while on the job (drug and alcohol use, and intentionally slow or sloppy work). Regardless of CWB′s many definitions, the activities stress the actual or potential harmful and detrimental effects of such behaviors on the organization and its members [9]. This study focuses on counterproductive work behavior, which is voluntary behavior that violates essential organizational and social norms, harming the organization and its shareholders and stakeholders (employers, supervisors, co-workers, and customers). CWB may include overt behavior, such as direct assault and theft, and covert behavior, such as deliberately failing to follow instructions or working incorrectly [9].

**Hypothesis** **1 (H1).** *Job insecurity has a positive effect on CWB*.

### 2.2. Mediating Effect of Moral Disengagement

Bandura [10] coined the term “moral disengagement” to characterize the ability to control one′s behavior, which is fundamentally self-regulating yet can be deliberately triggered. Moral disengagement consists of eight interconnected cognitive mechanisms, each of which permits people to disregard internal moral norms and act in ethically dubious ways. These mental processes help people feel better [11].

Moral disengagement is a form of social cognitive disengagement. Incorrect, dysfunctional, and antisocial conduct is mentally transformed through this procedure to detach it from these negative characteristics. Moral disengagement, in particular, releases the content and connotations generally connected with transgressive activity [12]. As a result, the offender is neither internally unpleasant nor hampered in pursuing their wants or goals by undertaking the act. Instead, individuals can use excuses or justifications to rationalize the harms and wrongs caused by their conduct.

In previous research, Probst et al. [13] investigated the individual and organizational antecedents and implications of security-related moral disengagement disclosure. They used resource protection, social exchange, and psychological contract breach as theoretical foundations, with a survey of 389 working adults from the United States. The findings show that job insecurity may have a role in employee morale.

Du and Zheng [14] investigated the mediating function of moral disengagement in the link between workplace exclusion and employees′ unproductive work behavior using social identity theory. Their findings revealed that: (1) workplace exclusion positively influenced workers′ counterproductive work behavior; and (2) moral disengagement entirely mediated the link between workplace exclusion and employees′ counterproductive work behavior.

Employees in the IT business affected by COVID 19 are dissatisfied with their job prospects. Low-skilled IT workers are at risk of layoff. As a result, employees are under additional pressure and face concerns about whether they will keep their positions. Moral disengagement may occur, and negative reciprocity may induce employees to deliver negative job results. In addition, employees may feel anxious because of the pressing need to improve their skills. As a result, work instability may impact CWB, with moral disengagement functioning as a mediating factor. Therefore, our research proposes the following hypothesis:

**Hypothesis** **2 (H2).** *Moral disengagement mediates the relationship between job insecurity and CWB*.

### 2.3. Moderated Mediating Effect of Psychological Capital

The term “psychological capital” refers to an individual′s positive psychological state of development, which means: (1) having the confidence (efficacy) to take on, and make the necessary effort to succeed at, challenging tasks; (2) making a positive attribution (optimism) about succeeding now and in the future; and (3) persevering toward goals and, when necessary, redirecting paths to achieve them. Olaniyan and Hystad [15] found a link between psychological capital and job insecurity among maritime industry workers.

Undoubtedly, psychological capital is a vital resource composed of self-efficacy, optimism, hope, and resilience. Self-efficacy refers to individuals′ task-specific self-confidence and the belief that they can achieve objectives effectively. It constitutes the individuals′ beliefs and abilities to succeed in specific conditions or complete task targets, including their beliefs in their capability to produce effects [16]. Optimism refers to expecting successful outcomes and reacting to issues with high confidence and personal abilities. In brief, optimism is individuals′ faith regarding their abilities to improve a situation [17]. Hope is an optimistic perspective and attitude of mind based on positive outcome expectations related to events and conditions in an individual′s life. People are hopeful of achieving a particular goal and having a plan for its attainment [18]. Finally, resilience refers to the ability to quickly recover when faced with difficulties [17]. These components are crucial factors that determine an individual′s behaviors. The possession of psychological capital relates to an individual’s achievements and wellbeing. Therefore, training and developing psychological capital will impact an enterprise′s existence, development, and prosperity [19].

The conservation of resources (COR) theory [20] asserts that employees with high personal resources will potentially cope with and control job-related stressors. Accordingly, we can consider individual differences as the capital for diminishing the stressors′ negative impacts on performance outcomes. Psychological capital [21], as an individual resource and positive psychological state, is significant to how employees interpret their environment, available resources, and resource constraints, and how they use the available resources to control the situation [22]. COR theory states that there is an actual loss of valuable resources when faced with a perceived threat of resource loss (e.g., job insecurity) or when the resources invested do not produce the expected return [23]. Job insecurity is a threatening or hindering stressor because employees tend to perceive it as an obstacle to personal growth and task accomplishment, triggering job stress (e.g., moral disengagement) [24]. In contrast, as a positive resource, psychological capital is predicted to positively moderate such threatening stressors.

**Hypothesis** **3 (H3).** *Psychological capital negatively moderates job insecurity and moral disengagement.*

According to moral disengagement theory [25], when one′s moral beliefs and values justify unethical behavior, there is less discord or inhibition in engaging in unethical behavior (e.g., counterproductive work behaviors) because one perceives this behavior as acceptable. Employees suffering from poor health effects due to job instability may perceive counterproductive work behavior as a legitimate form of response to the hardship they have endured [26]. People who lack job security are more inclined to see deviant behavior as a justifiable means of “getting back” at the organization and those in it for making their positions insecure, rather than seeing it as unethical [4,27].

According to the motivational process of the job demands-resources (JD-R) theory [28], as a work resource, psychological capital has natural motivational properties that can motivate employees and increase work engagement, which can have a positive impact [29,30,31]. In the present study, psychological capital as a work resource has motivational potential. Such a work resource can improve employees′ psychological states, thus improving their performance and reducing the harmful effects of moral disengagement on counterproductive work behaviors.

**Hypothesis** **4 (H4).** *Psychological capital negatively moderates moral disengagement and counterproductive work behavior*.

Individuals who have greater resources will cope better with stressful situations and vice versa [32,33]. Sora et al. [34] explored the relationships among objective and subjective job insecurity, job and collective self-efficacy, and affective wellbeing in employees from 138 Spanish and Austrian organizations. The findings consistently showed a negative relationship between subjective job insecurity and affective wellbeing. High self-efficacy is related to stress process regulation, higher self-esteem, better psychological and physical wellbeing, optimal adaptation to unpredictable situations, and high performance [35]. Zheng et al. [36] believe optimism is a buffer against job insecurity because optimists usually look toward future events with hope and positive expectations. The results of their quantitative study show that optimism significantly interacts with JI to predict job satisfaction and better job performance. There is a reinforcing action in job performance and satisfaction when job insecurity rises among highly optimistic employees [36]. Accordingly, we expect that optimistic employees will continue to work hard under uncertain employment situations, and actively deal with adversities and stress, because they believe in positive outcomes. Therefore, based on prior research, we predict that psychological capital will moderate the mediating effect of moral disengagement between job insecurity and counterproductive work behavior. A high level of psychological capital will weaken moral disengagement′s mediating effect.

**Hypothesis** **5 (H5).** *Psychological capital will moderate the mediating effect of moral disengagement between job insecurity and counterproductive work behavior. High psychological capital will weaken moral disengagement′s mediating effect*.

### 2.4. Moderated Mediating Effect of Negative Emotion

Negative emotions are character attributes that negatively impact personal emotions [37]. For example, the depression that develops naturally during personal development and the depression that many healthy people encounter is a negative emotion [38]. Thoughts, beliefs, expectations, decisions, and interpersonal relationships all impact the severity of depression. Inherited symptoms, such as anxiety and depression, can manifest as aggressive or counterproductive behaviors in youth, and these negative feelings can be exhibited as aggressive or counterproductive work behaviors. In addition, negative emotions represent various conditions related to disgust, including anger, hate, guilt, fear, and nervousness, and may also be displayed as mental distress or physiological abnormalities [39].

Job insecurity is a job-related stressor that adversely affects an individual′s mood and energy [40]. Lazarus [41] asserted that negative emotions may result from harm and threats to valuable outcomes. Job insecurity, due to its potential to harm personal growth or gains, triggers negative emotions [42]. Fida et al. [38] argue that negative emotions can activate moral disengagement as a secondary cognitive process, temporarily blurring an individual′s ethical standards, and enabling transgression as a reasonable behavioral coping strategy. Job insecurity can lead to stress, and humans naturally avoid situations that lead to chronic stress [43,44,45]. In short, employees with negative emotions are more likely to produce adverse organizational outcomes [46], which is in line with the JD-R theory′s “dual path” hypothesis. Excessive work demands can lead to employee exhaustion, which negatively impacts the organization [47].

**Hypothesis** **6 (H6).** *Negative emotions positively moderate job insecurity and moral disengagement*.

According to the stress model [48], the presence of one or more of these conditions is not sufficient to induce counterproductive work behavior. Instead, the underlying factor is the employee′s assessment of a situation, such as stress. When someone perceives a situation as stressful, it triggers negative emotions, which lead to aggressive behavior. In this sense, Spector′s model [49] emphasizes the role of negative emotions and influences the regulation of aggressive behavior, according to the traditional assumption that frustration and irritation may lead to harmful behavior [50]. Thus, emotions play a crucial role in work stress because they represent a response to the perceived stressful situation.

Employees with high levels of negative emotions tend to view the world more negatively [9]. Therefore, they may be more motivated to engage in behaviors that they believe will reduce or help them cope with their negative emotions [51]. Thus, the concept of “emotion management” [52] explains the relationship between negative emotions and CWB: employees experiencing negative emotions will seek to repair their emotional state through CWB. For example, employees who perceive the organization as the source of their negative emotions will seek revenge by acting negatively toward the organization. [53,54]. Another example of CWB is employees trying to repair their emotional state by avoiding problems [52,55]. Several studies examining the relationship between negative affect and CWB [56,57,58,59,60] found that individuals with high negative emotions are more likely to engage in CWB than those with low negative emotions. In addition, employees with higher negative affect tend to be more sensitive and emotionally reactive to their work experiences than employees with lower negative affect [61]. This greater reactivity can make individuals with high negative affect more likely to translate their emotions into CWB than individuals with low emotional reactivity.

**Hypothesis** **7 (H7).** *Negative emotions positively moderate moral disengagement and counterproductive work behavior*.

Some believe that emotions influence individual job performance through the affective event theory (AET) [62,63]. The theory explains that the surrounding environment influences a person′s internal state (e.g., cognitive and affective), affecting the person′s attitude, behavior, and performance [64]. If this condition exists in the organization, members of the organization react emotionally in various contexts. These emotional reactions then affect the individual′s attitudes and behaviors, which affect the individual′s performance. The role of emotion regulation in conflict resolution has long been studied [65]. In particular, negative emotions and conflict resolution approaches have been conducted primarily in political science and sociology. For example, scholars believe that accumulated negative emotions over time influence conflicts between ethnic or racial groups. Negative emotions in our study focus on whether emotional reactions resulting from job insecurity have an accelerator-like effect on one′s moral disengagement. Of course, if well controlled, negative emotions can effectively reduce conflict and its resulting damage [66].

**Hypothesis** **8 (H8).** *Negative emotions will moderate the mediating effect of moral disengagement between job insecurity and counterproductive work behavior. High negative emotions will strengthen the mediating effect.*

**Figure 1:**
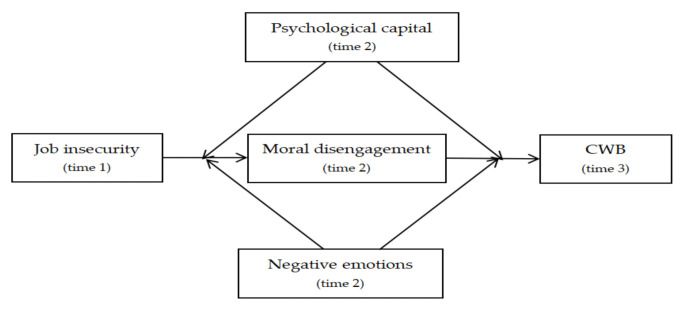
Hypothesized model.

Based on the above hypotheses, Figure 1 shows the research model.

## 3. Methods

### 3.1. Sample and Procedure

We collected data from eight Chinese IT companies located in Nanjing, Shanghai, and Nantong. Because all participants were Chinese, we translated the survey from English into Chinese [67]. We used a longitudinal study because these studies provide a complete view and key turning points in the development process. Another advantage of longitudinal studies is their suitability for studying the stability of development and the role of early influences, including case studies [68].

We collected data through online questionnaires at three points (baseline, three weeks, and six weeks). We contacted IT companies′ HR departments via email requesting permission to survey their employees at the three points; eight companies agreed. The first page of the questionnaire explained the purpose of our research and assured all respondents anonymity and confidentiality:

*“According to article 33 of the Statistics Law of the People′s Republic of China, this questionnaire is conducted anonymously, and the questionnaire does not involve the personal privacy or confidentiality of the enterprise. All the information you provide will be only used for academic research. All data obtained from participants will be confidential and will only be reported in an aggregated format to academic researchers without going through the company or a third one. In order to ensure the effectiveness of academic research, please feel free to fill it in according to your actual situation.”*

After the participants read and agreed to the content, they completed the questionnaire. We conducted the first survey on the job insecurity variable from 3 to 10 February. We received 553 valid responses. Then, we ran the second survey from 24 February to 1 March, measuring moral disengagement, negative emotions, and psychological capital. After eliminating invalid responses, we received a total of 469 questionnaires. The third survey was from 20 to 27 March and measured the counterproductive work behavior variable. At this stage, we had a total of 336 valid questionnaires. After matching the last four digits of the cell phone number in the first questionnaire with the last four digits of the cell phone number in the second and third questionnaires, we had 298 valid questionnaires. Thus, the retention rate was 53.89% (i.e., number of valid questionnaires finally collected/number of valid questionnaires received during the first survey).

### 3.2. Participants

We derived frequency statistics on personal information, including gender, age, education, working years, and position. There were 239 males (80.2%) and 59 females (19.7%). The participants′ ages were 20–25 (34.6% of total), 26–30 (30.9%), 31–35 (17.4%), and 36–40 (17.1%). Of the total participants, 50.6% had Master′s degrees, 41.9% had a Bachelor′s degree, and the remainder had other educational backgrounds. Their working experience was: 0–3 years (61.1% of total respondents), 3–5 years (25.5%), and those with five or more years of working experience accounted for 13.4%. Liepin′s 2020 IT Industry Talent Data Report (source: https://www.sohu.com/a/437272285_747398 (accessed on 27 July 2021)) states that China′s IT industry includes 81.7% men and 18.3% women. The proportion of 25–30 year old employees was the largest at 46.84%, followed by 30–35 (25.67%) and 20–25 (6.83%), thus indicating millennials are the main force in China′s IT industry. Based on Liepin′s information, our study′s data had a similar age and gender distribution as that of the IT industry in China in general.

### 3.3. Measures

#### 3.3.1. Job Insecurity

The COVID-19 pandemic presented companies with many obstacles in their daily management activities, including changing business needs. Previous studies found that job insecurity due to COVID-19 has a more significant impact on millennials. Therefore, our study adopted the four-item scale developed by Witte [69] to assess the degree of millennials′ job insecurity. Preliminary and formal investigations verified the scale′s reliability and validity with good endogenous adaptability. Measurement items included: “I will likely lose my job in my company very soon, and it makes me anxious” and “I am not sure I will be able to keep my job in my company.” We asked participants to respond to the statement using a five-point Likert scale ranging from 1 = *strongly disagree* to 5 = *strongly agree.*

#### 3.3.2. Moral Disengagement

The moral disengagement scale in this study used three items from Chen et al. [70]. We used a five-point Likert scale (1 = *strongly disagree* to 5 = *strongly agree*) for scoring all proactive questions. Items included: “In order to protect the company′s interests, it is okay not to adhere to the thorough truth”, “In order to protect the interests of the company, it is also possible to beautify the facts”, and “In order to protect the company′s interests, it is also possible to conceal information unfavorable to the company from the public”.

#### 3.3.3. Negative Emotion

We used five items from Spector and Fox [49] and Van Katwyk et al. [71] for the negative emotion scale. We scored all proactive questions using a five-point Likert scale (1 = *strongly disagree* to 5 = *strongly agree*). Sample items included: “My work makes me angry,” “My work makes me anxious”, “My job is annoying to me”, “My work makes me feel scared and horrified”, and “My work irritates me”.

#### 3.3.4. Psychological Capital

We adopted 16 items developed by Luthan and Youssef [72] to assess the degree of psychological capital. Preliminary and formal investigations verified the scale′s reliability and validity with good endogenous adaptability. Specific measurement items included: “I am confident in analyzing long-term problems to find solutions”, “I am confident in setting goals in my field of work”, and “I am confident in meeting outsiders to solve problems”.

#### 3.3.5. Counterproductive Work Behavior

This study′s counterproductive work behavior scale drew on 12 items from Bennett and Robinson [73]. We used a seven-point Likert scale for scoring: 1 = *never* to 7 = *daily*. Sample items were: “Have you taken property from work without permission in your company?”, “In your company, have you spent too much time fantasizing or daydreaming instead of working?”, and “Have you falsified a receipt to get reimbursed for more money than you spent on business expenses in your company?”.

#### 3.3.6. Control Variables

Previous research argued that gender impacts resilience. In addition, there may be a relationship between CWB and gender, age, position, and level of education [4]. Thus, we controlled for employee gender, age, education level, and work experience to minimize the possible effect of other variables not included (omitted variables) in the study.

## 4. Data Analysis and Results

### 4.1. Preliminary Analyses

We used Cronbach′s alpha to measure the questionnaire′s reliability. The larger Cronbach′s alpha coefficient, the higher the reliability and stability of the questionnaire. Because the results of Cronbach′s alpha coefficients of psychological capital, job insecurity, negative emotions, moral disengagement, and counterproductive work behavior in this study were higher than 0.8, the questionnaire scale in this study had good reliability [74].

The KMO value was 0.924, i.e., larger than 0.7, indicating that the employee questionnaire data is suitable for factor analysis. The factor analysis results showed a total explanatory power of 76.084%, i.e., greater than 50%. The factor loadings of each measurement item were all greater than 0.5, and each item fell into the corresponding factor, indicating the scale′s good structural validity [75]. The factors′ eigenvalues were more than 1, and the variance explained by the first principal factor was 36.313%, i.e., less than the critical criterion of 40%. Therefore, this study had no serious common method bias.

The main goal of confirmatory factor analysis (CFA) is to match the observed data with a specific factor structure. In this study, we constructed a structural equation model (SEM) for CFA using AMOS 24.0. The CFA equation can verify the corresponding relationship between scale items and unobservable variables. It can also detect the relationship between unobservable variables. In the study, the standard estimates (SE) for all items were higher than 0.50, and the *p*-value was less than 0.001, indicating that all items could accurately predict their variables. Furthermore, because all of the average variance extracted (AVE) values were higher than 0.50, i.e., 0.729, 0.647, 0.687, 0.676, and 0.732, the constructs had a high degree of convergent validity [76]. In addition, the comprehensive reliability (CR) value was higher than 0.70, indicating that all items had high reliability.

In this study, CMIN/DF was 1.606 (less than 3), SRMR was 0.069 (less than 0.08), CFI was 0.955 (more than 0.9), and RMSEA was 0.045 (less than 0.08). These results are consistent with the ideal values, indicating an acceptable model. Because there are multiple explanatory variable models in this study, we used SPSS25.0 to test for multicollinearity between variables. Hair et al. [77] showed that multicollinearity might exist if the tolerance of an independent variable is less than 0.1. In this study, the tolerance of all variables was higher than 0.1, indicating no multicollinearity between the variables. In addition, the VIF values of all variables were less than 5. This result further verifies no multicollinearity existed among all of the variables in this study. Table 1 shows the descriptive statistics and relationships among all variables.

### 4.2. Hypothesis Tests

Table 2 reports the significance testing results of the structural model path coefficients. As shown by the results, job insecurity has a significant positive effect on counterproductive work behavior (β = 0.612 ***). These results support Hypothesis 1, indicating that employees with higher job insecurity have higher counterproductive work behavior. In addition, moral disengagement as a mediating variable had a significant indirect effect on the relationship between job insecurity and CWB (β = 0.128 *). Therefore, the results support Hypothesis 2.

Table 3 shows that the interaction term between job insecurity and psychological capital significantly negatively affected moral disengagement (β = −0.240 *). This result indicates that psychological capital negatively moderated the effect of job insecurity on moral disengagement. Therefore, this finding supports Hypothesis 3. Furthermore, the interaction term “int” between psychological capital and moral disengagement significantly negatively affected CWB (β = −0.295 **), supporting Hypothesis 4. Our results also support Hypothesis 6: the interaction term (int) between job insecurity and negative emotions had a significant positive effect on moral disengagement (β = 0.408 **), indicating that negative emotion, as a moderating variable, positively moderated the effect of job insecurity on moral disengagement. However, our results did not support Hypothesis 7. We did not find a significant positive effect on CWB (β = 0.162) in the interaction between negative emotions and moral disengagement.

Next, we used model 58 in Process macro 3.5 to test whether PC and NE can moderate the multiple mediation effects in the relationship between job insecurity and CWB; we used SPSS 25.0 to run the calculations. After constructing the moderated multiple-mediation model with job insecurity as the independent variable, CWB as an outcome variable, moral disengagement as a mediator, and PC and NE as moderators, we inserted all of the control variables into the model. Following this, we set the number of bootstrap samples to 5000. Then we used the bias-corrected bootstrapping method to conduct the test at a 95% confidence level.

Table 4 reports the moderated mediation effect between job insecurity and CWB moderated by psychological capital. As shown in Table 4, we found a significant difference in the indirect effects of moral disengagement between job insecurity and CWB at different levels of PC. The bootstrap 95% confidence interval of the mediation′s difference (β = −0.0543) not containing 0 (−0.1172 ~ −0.0531) indicates that PC has a significant moderating effect on the mediation of moral disengagement between job insecurity and CWB. When psychological capital is high, the mediating effect is weak. This finding supports Hypothesis 5.

Table 5 reports the moderated mediation effect between job insecurity and CWB moderated by negative emotions. Table 5 shows a significant difference in the indirect effects of moral disengagement between job insecurity and CWB at different NE levels. The bootstrap 95% confidence interval of mediation′s difference (β = 0.0436) not containing 0 (0.0129~0.1583) means that NE has a significant moderating effect on the mediation of moral disengagement between job insecurity and CWB. Thus, there is a strong mediating effect when negative emotions are high. This result supports Hypothesis 8.

### 4.3. Robustness Tests

This study tested the robustness of the results by conducting repeated regression analyses for counterproductive work behavior (see Table 6). We analyzed job insecurity (β = 0.493 ***) on counterproductive work behavior (Models 1 and 2), supporting Hypothesis 1. Our analysis of job insecurity (β = 0.432 ***) on moral disengagement (Model 3) and moral disengagement (β = 0.188 ***) on CWB (Model 4) supported Hypothesis 2. The results of these four models are qualitatively identical to our main analyses with SEM (structural equation modeling), confirming the robustness of our main analyses. We confirmed all hypotheses supported in the main analyses with regression analyses.

This study also tested a conceptual model by adding two moderating variables in a separate model—psychological capital and negative emotions. We found that the interaction term “int” between job insecurity and psychological capital (β = 0.248 *) had a significant negative effect on moral disengagement (Models 5 and 6). The interaction term (int) between moral disengagement and psychological capital (β = −0.258 *) had a significant negative effect on CWB (Models 7 and 8). The interaction term (int) between job insecurity and negative emotions (β = 0.345 **) had a significant positive effect on moral disengagement (Models 9 and 10). However, we found no significant positive effect on moral disengagement (Models 11 and 12) and CWB (β = 0.124). These findings support Hypotheses 3, 4, and 6, but not Hypothesis 7. Furthermore, these analyses generated identical results for our hypothesis tests, indicating the robustness of the conclusions.

**Figure 2:**
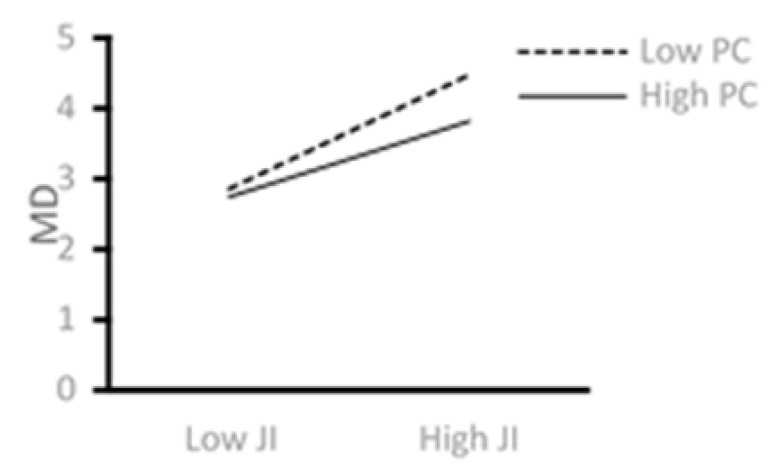
The moderating role of PC.

Figure 2 shows that higher levels of job insecurity are associated with higher levels of moral disengagement. However, job insecurity′s effect on moral disengagement decreases when IT employees have a more heightened sense of psychological capital, thus supporting Hypothesis 3.

**Figure 3:**
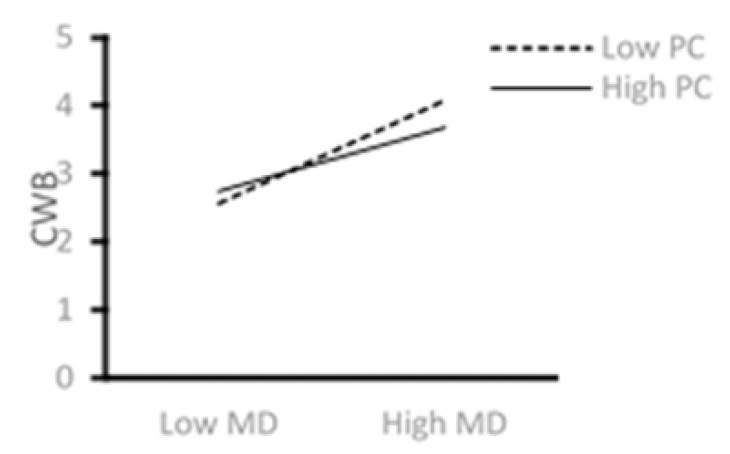
The moderating role of PC.

Figure 3 shows an association between higher levels of moral disengagement and higher levels of CWB. However, there is a reduced effect of moral disengagement on CWB when IT employees have a higher sense of psychological capital. This result supports Hypothesis 4.

**Figure 4:**
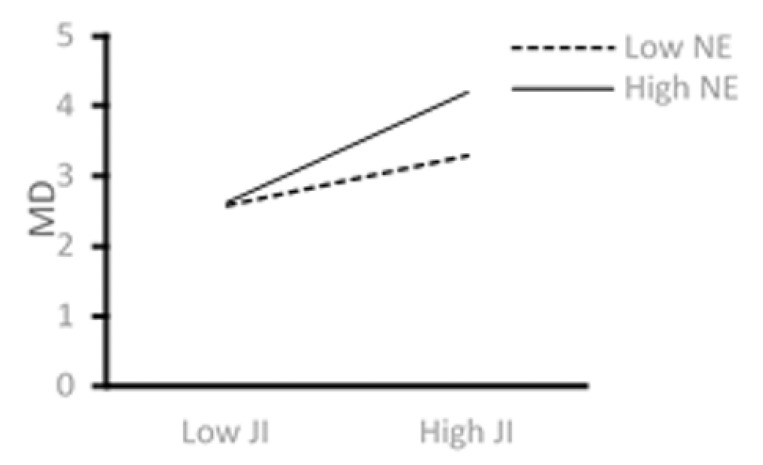
The moderating role of NE.

Figure 4 shows higher levels of job insecurity associated with higher levels of moral disengagement. There is an increased effect of job insecurity on moral disengagement when IT employees have a greater sense of negative emotions. This outcome supports Hypothesis 6.

## 5. Discussion

This study investigated the effect of job insecurity on counterproductive work behavior (CWB) based on COR theory and affective event theory. In addition, we developed a relationship model using moral disengagement as a mediating variable between job insecurity, moral disengagement, and counterproductive work behavior. Finally, we used negative emotions and psychological capital as moderating variables to explore the moderating role of negative emotions and psychological capital in the relationship between job insecurity, moral disengagement, and counterproductive work behavior.

In this study, 298 millennials working in Chinese IT companies participated in the survey. We collected and measured data from the same employees during three different time intervals to reduce common method bias and mitigate the issue of single-source data. First, we measured job insecurity at time 1. Then, after a time lag of three weeks, we measured negative emotions, moral disengagement, and psychological capital. Following this, we measured counterproductive work behavior six weeks after baseline. We used SPSS25.0 and Amos24.0 for structural modeling and data analysis.

This research indicated that, first, there is a positive association between job insecurity and counterproductive work behavior. Second, moral disengagement played a mediating role. This finding means that the threat of job insecurity in an organization can increase moral disengagement, leading to behaviors that harm the organization, such as counterproductive work behaviors. Third, we verified psychological capital′s moderating role. Psychological capital moderated the relationship between job insecurity, moral disengagement, and counterproductive work behavior. A high level of psychological capital weakens the mediating effect. Fourth, negative emotions have a positive moderating effect between job insecurity and moral disengagement. Negative emotions also moderated the mediating effect of moral disengagement between job insecurity and CWB. However, in this study, negative emotions did not have a positive moderating effect between moral disengagement and CWB. This result may be due to the generally high stress levels and negativity among employees in the COVID-19 environment.

Some theoretical and practical implications are as follows.

First, as explained in social exchange theory, an employee′s organization does not provide job security. Employees may feel helpless in the absence of essential resources, such as workforce benefits, which may trigger negative behaviors. Morf et al. [78] demonstrated that CWB is a coping behavior exhibited by employees under unsatisfactory work conditions, including uncertainty about future work. Our research extends the study of job insecurity to the IT industry, focusing on the counterproductive work behavior of IT employees, thereby expanding the scope of the study. Moreover, existing CWB studies primarily focus on employees in the traditional industrial environment, ignoring the new characteristics of CWB among knowledge workers. Knowledge workers often possess knowledge resources that are crucial to enterprise development or innovation, and their CWB may be more harmful. Therefore, this study has some theoretical significance.

Second, the study results concluded that moral disengagement plays a partial mediating role in the relationship between job insecurity and Chinese IT employees′ counterproductive work behavior. Consistent with the predictions, our results support the research hypotheses. The findings show that job insecurity increases employees′ moral disengagement, leading to counterproductive work behavior. These observations are consistent with Huang et al.′s [4] empirical study.

Third, the results of this study confirm that negative emotions partially moderated the relationship between job insecurity and moral disengagement. Job insecurity causes employees to become stressed. According to the theoretical framework of the stressor-emotion model, the more that employees are negative about stress, the more they are morally relaxed. These findings are also consistent with the investigations of Fida et al. [38].

Fourth, we introduced psychological capital as a moderating variable to explore its mechanism in job insecurity and Chinese IT employees′ counterproductive work behavior. Previous research found that psychological capital, as a positive internal psychological state, may also serve as a moderator of job insecurity. Probst et al. [13] focused more on constructing positive external supportive relationships, somewhat ignoring the underlying internal psychological mechanisms. Given that internal and external factors can jointly mitigate the harmful effects of job insecurity, this study introduced psychological capital as a moderating variable, and the results confirm that psychological capital negatively moderates job insecurity, moral disengagement, and counterproductive work behavior.

It is difficult for organizations to provide a high level of job security for millennial employees in a rapidly evolving, volatile, and highly competitive climate, especially in an emerging economy such as China. As a result of these findings, this research makes some recommendations for reducing job insecurity and counterproductive work behavior among millennial IT employees.

First, organizations should use training and instructional interventions to enable employees to cope effectively with stress. For example, the Psychological Capital Intervention Model [79] is a psychological capital-building educational model. According to this model, psychological resilience can be developed via face-to-face preparation, resulting in increased employee effectiveness [21]. In addition, managers can enhance their employees′ psychological resources [80], improve millennial employees′ mental health and performance, and reduce negative employee attitudes by using training programs and service providers [81].

Second, by providing unique training programs and employing modern leadership tactics, management teams will boost their employees′ positive views. They can start by familiarizing staff with the organization′s aims and emphasizing their importance in accomplishing those goals [82]. Then, it is critical to identify and educate people about their positive traits and skills (many employees have no idea of their capabilities) to develop psychological capital—teaching employees how to build and strengthen positive innate features is beneficial. Using adverse event analysis methodologies, managers can enhance employee optimism by focusing on good outcomes.

Third, given that employees′ CWB is harmful to the organization, employers must minimize this behavior. Managers should find ways to enhance employees′ perceptions of job security, keep explicit promises, and avoid layoffs. If the organization can no longer provide secure jobs, it should inform its employees honestly and early.

Despite its numerous theoretical and methodological strengths, the current study is not free from limitations. For example, although we collected time-lagged data from Chinese IT employees at different time intervals, this study is not a pure longitudinal study. In addition, the uniqueness of the data collected during the COVID-19 pandemic may be problematic. Furthermore, the distinct data contribution and the collection period make it difficult to generalize the results. Future studies should use a complete cross-lagged panel design to reproduce our results, gathering data on all variables at different periods. Although we ensured the anonymity and confidentiality of the questionnaire for the participants, the data were collected using a self-reported survey instrument. Finally, given the sensitivity of CWB, respondents may not have answered according to their actual situation. Future research can allow superiors or colleagues to evaluate employees′ CWB more objectively.

## Figures and Tables

**Table 1:** Means, standard deviations, and correlations.

	M	SD	1	2	3	4	5	6	7	8	9
1.Age	28.59	5.65									
2.Work experience	2.67	1.40	−0.027								
3.Gender	1.20	0.40	0.052	0.008							
4.Education	2.55	0.63	−0.049	−0.014	−0.076						
5.PC	3.64	0.83	0.027	0.012	−0.067	0.010	**0.836**				
6.NE	2.17	0.87	−0.026	−0.056	−0.035	−0.087	−0.259 **	**0.822**			
7.JI	3.74	0.96	−0.027	0.043	−0.018	−0.002	−0.462 **	0.448 **	**0.854**		
8.MD	3.80	10.07	−0.012	0.017	−0.065	−0.043	−0.236 **	0.387 **	0.433 **	**0.804**	
9.CWB	4.69	10.53	0.012	−0.076	−0.082	−0.010	−0.477 **	0.335 **	0.482 **	0.360 **	**0.829**

Note: ** *p* < 0.01. The diagonal value (in bold) is the square root of the average variance extracted (AVE). *n* = 298, JI = job insecurity, NE = negative emotions, MD = moral disengagement, CWB = counterproductive work behavior, PC = psychological capital.

**Table 2:** Testing results of the structural model.

	Path	Estimate	S.E.	*p*	Result
H1	Job insecurity → Counterproductive work behavior	0.612	0.081	***	Accepted
H2	Job insecurity → Moral disengagement → Counterproductive work behavior	0.128	0.045	*	Accepted

* *p* < 0.05. *** *p* < 0.001.

**Table 3:** Bootstrap results for the moderating effects.

Effects			Estimate	S.E.	C.R.	*p*	Result
MD	<---	JI	0.293	0.177	4.638	***	Accepted
	<---	PC	−0.350	0.198	−2.862	**	
	<---	JI*PC(int)	−0.240	0.128	−2.326	*	
CWB	<---	MD	0.381	0.216	1.986	*	Accepted
	<---	PC	−0.461	0.288	−3.582	***	
	<---	MD*PC(int)	−0.295	0.227	2.536	**	
MD	<---	JI	0.781	0.158	4.931	***	Accepted
	<---	NE	0.382	0.160	2.853	**	
	<---	JI*NE(int)	0.408	0.155	2.346	**	
CWB	<---	MD	0.857	0.205	4.181	***	Not accepted
	<---	NE	0.544	0.233	2.080	*	
	<---	MD*NE(int)	0.162	0.08	1.583	0.072	

Note: * *p* < 0.05. ** *p* < 0.01. *** *p* < 0.001. *n* = 298, JI = job insecurity, NE = negative emotions, MD = moral disengagement, CWB = counterproductive work behavior, PC = psychological capital.

**Table 4:** Bootstrap results for the moderated mediation effect analysis of PC.

Mediating Path	PC	Effect	S.E.	95%CI		*t*
				LLCI	ULCI	
JI-MD-CWB	Low (Mean − 1 SD)	0.1695	0.0483	0.0822	0.2362	2.988 ***
	Mid (Mean)	0.1152	0.0407	0.0573	0.1802	2.384 **
	High (Mean + 1 SD)	0.0831	0.0388	0.0336	0.1256	1.847 *
	Difference	−0.0543	0.0338	−0.1172	−0.0531	−1.912 *

Note: * *p* < 0.05. ** *p* < 0.01. *** *p* < 0.001. *n* = 298, MD = moral disengagement, PC = psychological capital, CWB = counterproductive work behavior.

**Table 5:** Bootstrap results for the moderated mediation effect analysis of NE.

Mediating Path	NE	Effect	S.E.	95%CI		*t*
				LLCI	ULCI	
JI-MD-CWB	Low (Mean − 1 SD)	0.1253	0.0588	0.0466	0.1930	2.630 **
	Mid (Mean)	0.1689	0.0652	0.0864	0.3611	3.288 ***
	High (Mean + 1 SD)	0.2030	0.0538	0.1120	0.3986	3.842 ***
	Difference	0.0436	0.0531	0.0129	0.1583	2.346 **

Note: ** *p* < 0.01. *** *p* < 0.001. *n* = 298, JI = job insecurity, NE = negative emotions, MD = moral disengagement.

**Table 6:** Regression analysis for CWB and MD: model summary.

Predictors	Model 1 CWB	Model 2 CWB	Model 3 MD	Model 4 CWB	Model 5 MD	Model 6 MD	Model 7 CWB	Model 8 CWB	Model 9 MD	Model 10 MD	Model 11 CWB	Model 12 CWB
Age	0.014	0.027	0.000	0.026	−0.008	0.005	0.005	0.009	−0.003	−0.005	0.008	0.013
Work experience	−0.076	−0.097	−0.002	−0.097	0.012	0.005	−0.080	−0.011	−0.001	−0.002	−0.080	−0.087
Gender	−0.076	−0.068	−0.061	−0.057	−0.053	−0.062	−0.055	−0.062	−0.055	−0.049	−0.048	−0.050
Education level	−0.017	−0.015	−0.046	−0.007	−0.044	−0.043	−0.009	−0.020	−0.044	−0.050	−0.005	−0.002
JI		0.493 ***	0.432 ***	0.412 ***	0.420 ***	0.275 ***			0.481 ***	0.498 ***		
MD				0.188 **			0.283 ***	0.325 **			0.458 ***	0.608 ***
PC					−0.241 **	−0.265 **	−0.396 ***	−0.490 ***				
NE									0.319 **	0.342 **	0.329 *	0.425 *
JI × PC						−0.248 *						
MD × PC								−0.258 *				
JI × NE										0.345 **		
MD × NE												0.124
R 2	0.012	0.255	0.193	0.283	0.229	0.240	0.322	0.418	0.254	0.296	0.152	0.162
Adjusted R 2	0.000	0.242	0.180	0.268	0.221	0.228	0.315	0.395	0.248	0.280	0.134	0.142

* *p* < 0.05. ** *p* < 0.01. *** *p* < 0.001. *n* = 298, JI = job insecurity, NE = negative emotions, MD = moral disengagement, CWB = counterproductive work behavior, PC = psychological capital.

## Data Availability

The data used in this research are available on request from the corresponding author. The data are not publicly available due to restrictions, i.e., privacy or ethics.
